# Supplementary material for: Natural oxidase-mimicking copper-organic frameworks for targeted identification of ascorbate in sensitive sweat sensing
Source: Nat Commun. 2023 Jan 5;14:69. doi: 10.1038/s41467-022-35721-4 (PMC9814535; doi:10.1038/s41467-022-35721-4)
Supplement: Supplementary file 1 — Supplementary Information [file 41467_2022_35721_MOESM1_ESM.pdf]

Supplementary Information

Natural Oxidase-Mimicking Copper-Organic Frameworks for Targeted Identification of Ascorbate in Sensitive Sweat Sensing

Zhengyun Wang[1], *Yuchen Huang*[2], *Kunqi Xu*[3], *Yanyu Zhong*[1], *Chaohui He*[1], *Lipei Jiang*[1], *Jiankang Sun*[1], *Zhuang Rao*[1], *Jiannan Zhu*[1], *Jing Huang*[1], *Fei Xiao*[1], *Hongfang Liu*[1,*] and *Bao Yu Xia* [1,*]

[1] Hubei Key Laboratory of Material Chemistry and Service Failure, Key Laboratory of Material Chemistry for Energy Conversion and Storage (Ministry of Education), Hubei Engineering Research Center for Biomaterials and Medical Protective Materials, School of Chemistry and Chemical Engineering, Huazhong University of Science and Technology, 1037 Luoyu Rd, Wuhan 430074, PR China

[2] Secretariat license de chimie, bâtiment 460, Université Paris-saclay, 91400, Orsay, Paris, France

[3] Key Laboratory of Inorganic Functional Materials and Devices, Shanghai Institute of Ceramics, Chinese Academy of Sciences, Shanghai 201899, PR China

*Corresponding Author: liuhf@hust.edu.cn (H. Liu); byxia@hust.edu.cn (B. Y. Xia)

Experimental Section:

1. Materials

Acorbate, lactate, uric acid, glucose, ethanol, cortisol, tryptophan and histidine were purchased from Sigma–Aldrich Chemical Co. The ligand 5-ethoxyisophthalic acid was purchased from Jilin Zhongkeyanshen Technology Co., Ltd., and all the chemicals were analytical grade and used without any further purification.

2. Fabrication of ascorbate oxidase-based electrode

The ascorbate oxidase (AOx)-based electrode was fabricated according to the following procedure. Ten microliters of a mixture of commercial (250 IU) AOx/bovine albumin (BSA) (both 10 mg/mL) in 0.1 M phosphate-buffered saline (PBS) was prepared for drop casting on the carbon paper working electrode and dried under ambient conditions. AOx and BSA were the recognition species and stabilizer, respectively. After that, this surface was covered with 5 μL of 1 wt % chitosan solution prepared in 1 wt % acetic acid. Then, 0.5 μL of glutaraldehyde (0.5%) was cast on the AOx-based electrode for cross-linking. The AOx immobilized electrode was placed in a refrigerator at 4 ℃ overnight.

3. Characterization

X-ray diffraction (XRD) patterns were recorded using a diffractometer (X'Pert PRO, Panalytical B.V., Almelo, The Netherlands) equipped with a Cu-Ka radiation source (λ=1.5406 Å). X-ray photoelectron spectroscopy (XPS) measurements were performed on a Kratos-Axis spectrometer with monochromatic Al-Ka (1486.71 eV) X-ray radiation (15 kV and 10 mA) and a hemispherical electron energy analyser. The morphologies and structures of the materials were examined by field-emission scanning electron microscopy (FSEM, Nova Nano SEM 450) and transmission electron microscopy (TEM: Talosf200s). The Fourier transform infrared spectra of samples for amino acid functionalization were examined by an attenuated total reflectance infrared spectrophotometer (Nicolet iS5) and atomic force microscopy-based infrared spectrophotometer (AFM-IR). The Trp/His loading amounts were measured by liquid chromatograph mass spectrometer (LC-MS) (Ultimate 3000 UHPLC-Q Exactive) using methanol as the mobile phase . In situ electrochemical infrared spectroscopy (in situ EC-IR) of the

samples was recorded by a Fourier transform infrared spectrometer (Nicolet iS50R). The binding behavior of the samples was tested by isothermal titration calorimetry (ITC) (iTC200iTC200) at 25 ℃. The electrooxidation product ascorbate was examined with high-resolution mass spectrometry (HRMS, microTOF Ⅱ). Electrochemical performances were tested by an electrochemical workstation (CHI760E).

4. Electrochemical measurement

The electrochemical performance test was performed with a three-electrode system: carbon paper supported STAM-17-OEt or HT-STAM-17-OEt as the working electrode, Ag/AgCl as the reference electrode, and Pt as the counter electrode. The carbon paper electrode area is 0.23758 cm^{-2}. HT-STAM-17-OEt (4 mg mL^{-1}) was dispersed in 200 μL of H_2O as the ink for coating the carbon paper electrode. For the human sweat sensing platform, polytetrafluoroethylene (PTFE) was used as the flexible substrate to support the three-electrode system. The concentration of interferents used in the experiment refers to its maximum concentration in human sweat.

5. HT-STAM-17-OEt-based electrochemical sweat sensing platform

The preparation procedure of the electrochemical sensing platform device is as follows. Polyethylene terephthalate (PET) as the substrate (3 cm×5 cm) was selected for supporting electrodes. Carbon paper electrodes were tailored and pasted on the PET. The polyvinyl butyral (PVB) polymer reference electrode was fabricated according to the reported work.[1] PVB (400 mg), AgCl (20 mg), and KCl (20 mg) were placed in 5 ml methanol to obtain a homogeneous solution. After that, the mixture was exposed to a light source for 5 min to induce a partial reduction of AgCl. The mixture (500 μL) was dripped on a circular carbon electrode (diameter of 5.5 mm) and dried. HT-STAM-17-OEt and Pt were dripped and fixed on the circular carbon electrode. After drying all the electrodes and materials, the sensing device is completed. The cost of the sweat sensor was evaluated according to the major electrode material cost. The costs of the MOF ligand, AgCl reference and Pt counter electrode are 1300, 350, and 1200 RMB/g, respectively. The masses of ligand, AgCl, and Pt for the sweat sensor are 0.6 mg, 2 mg and 5 mg, respectively. Thus, the cost of a single sweat sensor is 7.5 RMB (0.8 RMB for HT-

STAM-17-OEt, 0.7 RMB for Ag/AgCl, 6 RMB for Pt). The first author, Zhengyun Wang, as a volunteer, was recruited for collecting sweat in the study, and informed consent was obtained from the individual.

6. Density functional theory calculation

First-principles calculations were performed within the framework of density functional theory (DFT). The linear combination of the atomic orbital method and generalized gradient approximation GGA/PW91 functional were employed. Double numerical plus polarisation (DNP) was employed as the basis set. The core electrons were obtained using the all-electron method considering all electrons in the system. The DFT+D method within the OBS scheme was adopted owing to the van der Waals (vdW) weak interaction. The self-consistent field (SCF) tolerance was set as 1×10^{-5} Hartree. For geometric optimization, the DFT calculations were performed at a spin-unrestricted set. The amino acids and Cu paddle wheel atoms were free, and the ligand atoms were constrained.

7. Finite element analysis

The secondary current distribution was used as the physical field to simulate the current density distribution on the surface of the micro/nanostructures of STAM-17-OEt and HT-STAM-17-OEt. To simplify the finite element analysis for micro/nanostructures, the geometry of the electrolyte was abstracted as a cylinder (radius: 20 μm, height: 10 μm), which completely includes the cuboid. The cylinder surface was set as the cathodic electrode, and the 3D cuboid surface was set as the anodic electrode. The electrode kinetic equations for cathodic and anodic electrodes are the Butler-Volmer equations and concentration-dependent Butler-Volmer equations, respectively. The boundary condition parameters for Figure 5G and Figure 5H are included in Table S3.

Figure and Captions

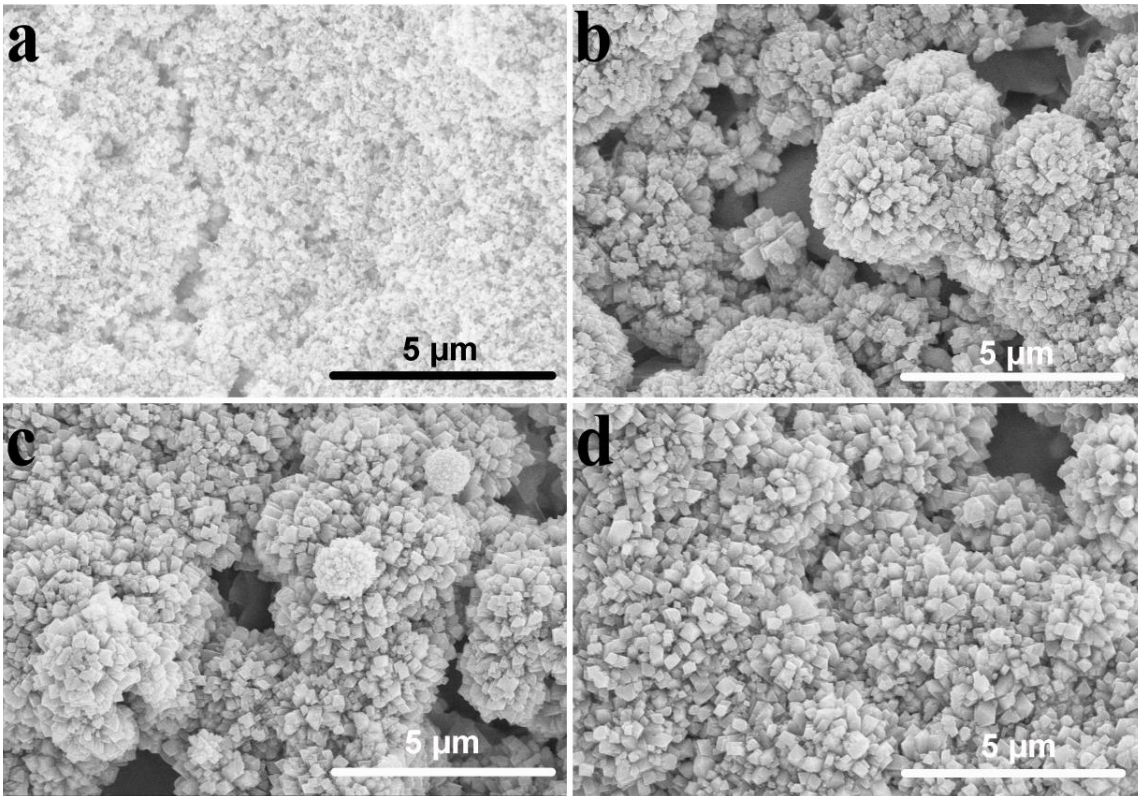

Supplementary Fig 1. FESEM images of prepared $Cu_2(OH)_3Cl$ by using $CuCl_2$ aqueous solution for hydrolysis with different concentrations of (a) 1 M, (b) 0.2 M, (c) 0.1 M, (d) 0.05 M.

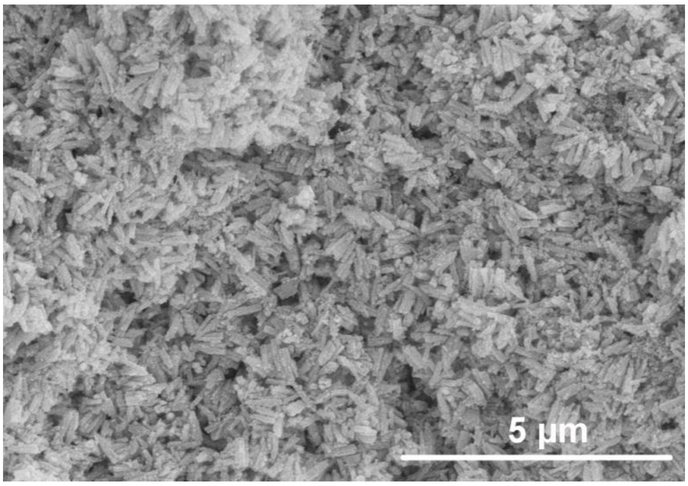

Supplementary Fig 2. FESEM image of prepared $Cu_2(OH)_3Cl$ nanocuboids by using $CuCl_2$ aqueous solution for hydrolysis with a concentration of 0.5 M.

To achieve $Cu_2(OH)_3Cl$, $CaCO_3$ paper can react with $H^{+,}$ which is produced by the hydrolysis of $CuCl_2$. The slow hydrolysis reaction process is as follows:

$$2Cu^{2+}+Cl^{-}+3H_2O \rightarrow Cu_2(OH)_3Cl+3H^{+} \qquad \text{s-1}$$

$$CaCO_3+2H^{+} \rightarrow Ca^{2+}+CO_2+H_2O \qquad \text{s-2}$$

Interestingly, we discovered that using $CuCl_2$ aqueous solutions with different concentrations would lead to different structures of $Cu_2(OH)_3Cl$ on $CaCO_3$ paper (Figure S1 and S2). When the concentration is relatively high (1 M), the obtained $Cu_2(OH)_3Cl$ shows a small size (Figure S1A). When the concentration of the $CuCl_2$ aqueous solution is relatively low (0.2, 0.1 and 0.05 M), the obtained $Cu_2(OH)_3Cl$ particles show larger and irregular sizes (Figure S1b-S1d). When the concentration is moderate (0.5 M), $Cu_2(OH)_3Cl$ shows a relatively uniform morphology of nanocuboids (Figure S2). Furthermore, we discovered that the STAM-17-OEt crystal shape is related to the morphology of $Cu_2(OH)_3Cl$. Using these irregularly sized $Cu_2(OH)_3Cl$ to react with 5-ethoxyisophthalic acid resulted in irregularly sized STAM-17-OEt crystals (Figure S3). STAM-17-OEt crystals with regular microslate morphology have been selected for amino acid modification to avoid performance differences caused by irregular sizes.

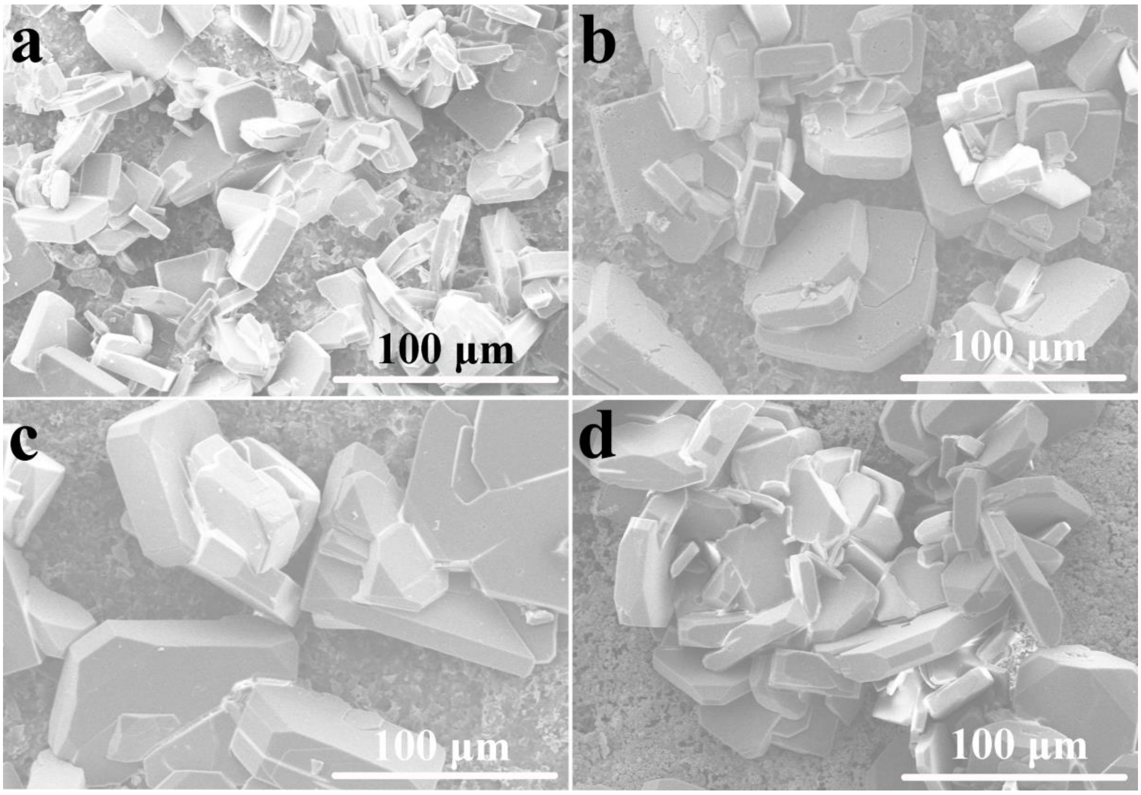

Supplementary Fig 3. FESEM images of STAM-17-OEt crystals prepared by using (a) 1 M, (b) 0.2 M, (c) 0.1 M, (d) 0.05 M $CuCl_2$ hydrolyzed $Cu_2(OH)_3Cl$ as precursors.

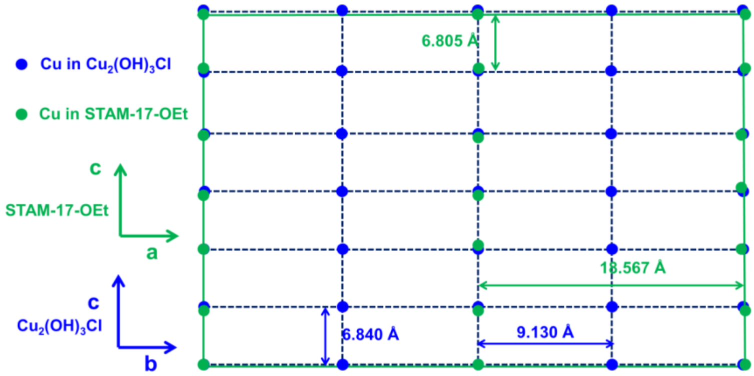

Supplementary Fig 4. Schematic illustration showing the crystal lattice of $Cu_2(OH)_3Cl$ and STAM-17-OEt from bc and ac projections, respectively.

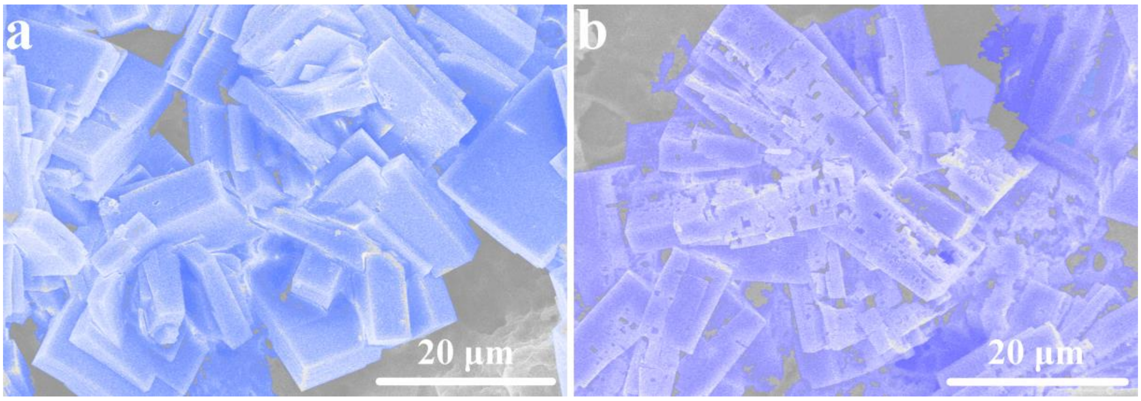

Supplementary Fig 5. FESEM images of (a) STAM-17-OEt and (b) HT-STAM-17-OEt.

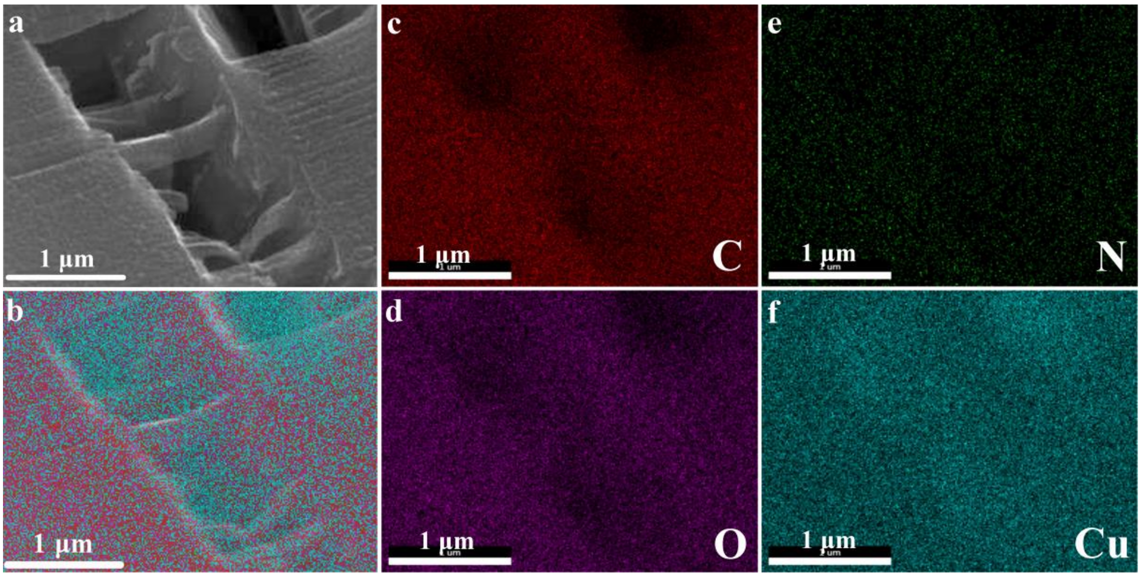

Supplementary Fig 6. (a-b) FESEM images of cubic defects on HT-STAM-17-OEt and corresponding overlap elementary mapping. (c-f) Corresponding elementary C, O, N, Cu mapping images of HT-STAM-17-OEt.

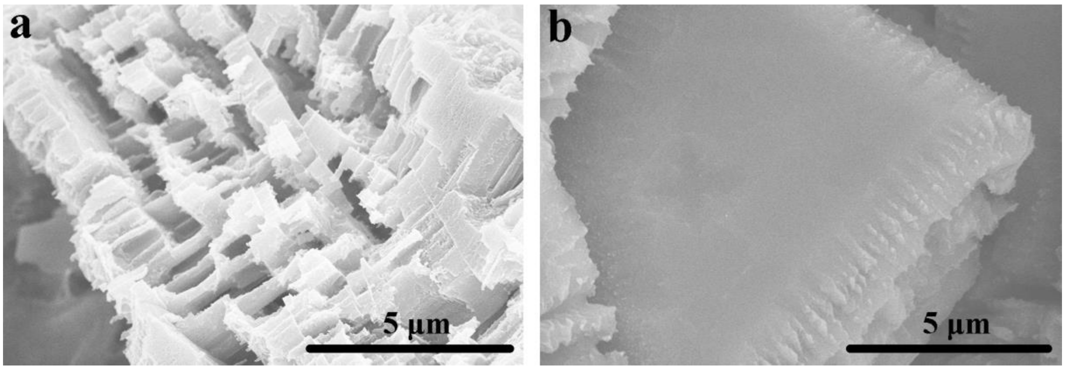

Supplementary Fig 7. (a) FESEM image of HT-STAM-17-OEt by using highly concentrated mixed tryptophan and histidine (50 mM for tryptophan and 25 mM for histidine) to react with STAM-17-OEt. (b) FESEM image of HT-STAM-17-OEt by using low concentrated mixed tryptophan and histidine (0.5 mM for tryptophan and 0.25 mM for histidine) to react with STAM-17-OEt.

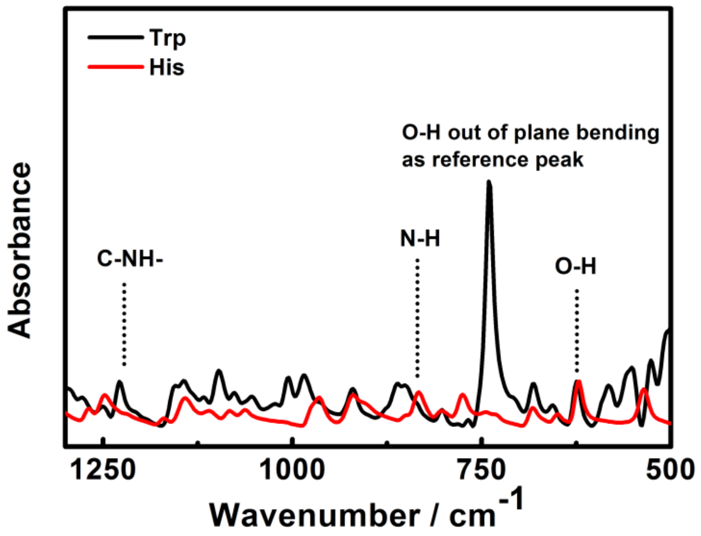

Supplementary Fig 8. ATR-IR spectra of Trp and His.

The ratio of the infrared absorbance coefficient for C-NH- (stretching)/N-H (in-plane rocking vibration) in Trp and His can be calculated as 1.3 by normalization of the common peak (O-H out-of-plane bending) in Trp and His. The absorbance ratio of C-NH- (stretching)/N-H (in-plane rocking vibration) in HT-STAM-17-OEt was further calculated to be 2.44.

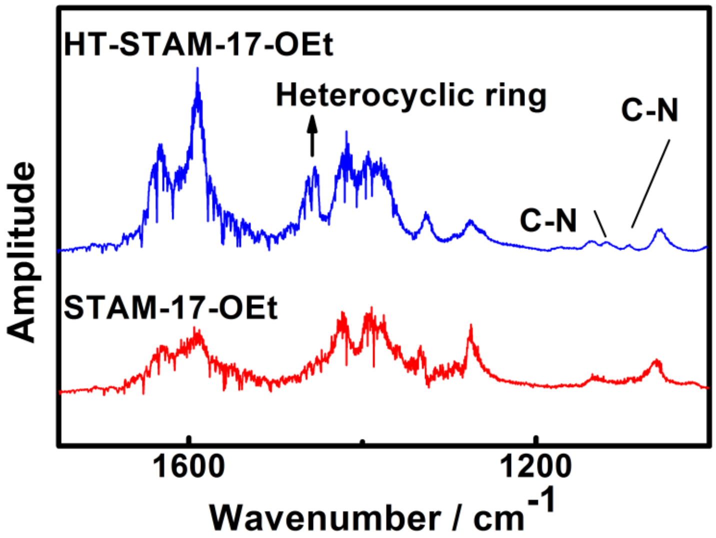

Supplementary Fig 9. AFM-IR spectra for single slate of STAM-17-OEt and HT-STAM-17-OEt. The peaks at 1466 and 1090 cm^{-1} correspond to heterocyclic ring stretching and C-N stretching in Trp, respectively. The peaks at 1462, 1453, 1118, and 1090 cm^{-1} are attributed to heterocyclic ring stretching and C-N stretching in His, respectively.

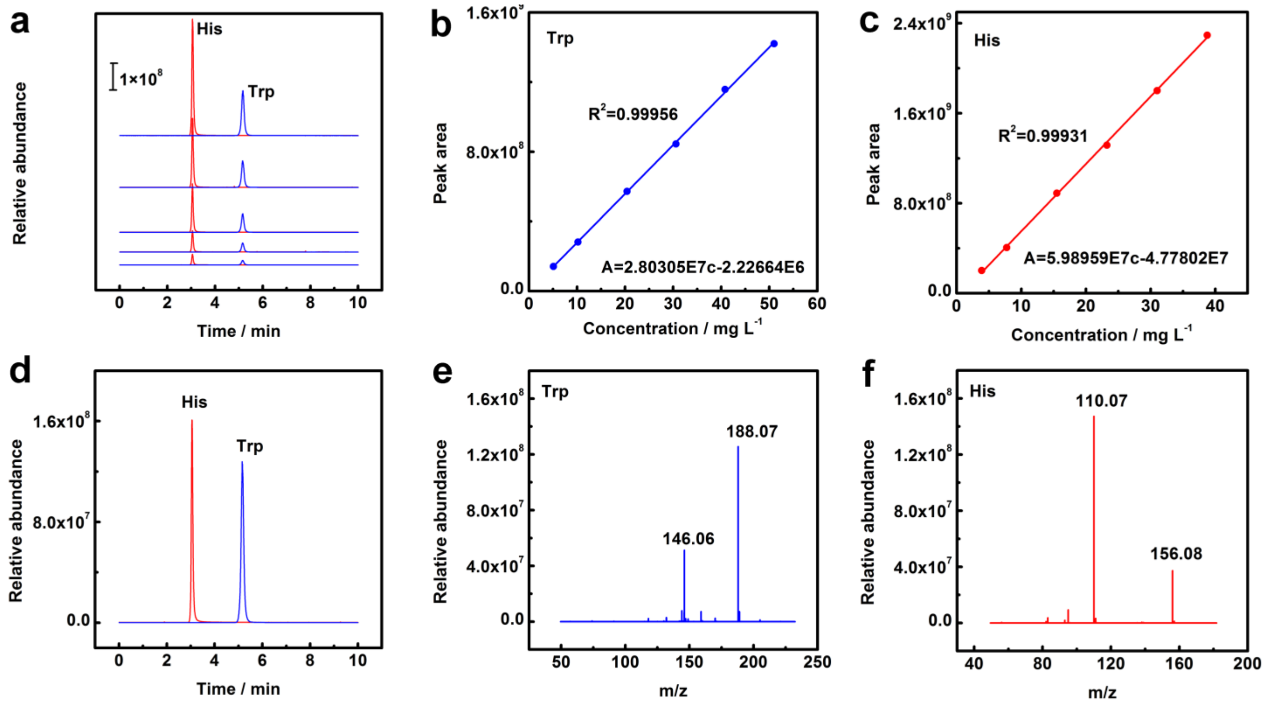

Supplementary Fig 10. (a) Liquid chromatograph for Trp and His with standard concentrations and (b-c) corresponding standard curves. The retention time of His and Trp is 3.05 and 5.16 min, respectively. (d) Liquid chromatograph of the remaining Trp and His with 20 times of dilution by dissolving STAM-17-OEt and removing other constituents. Mass spectrogram of separated (e) Trp and (f) His.

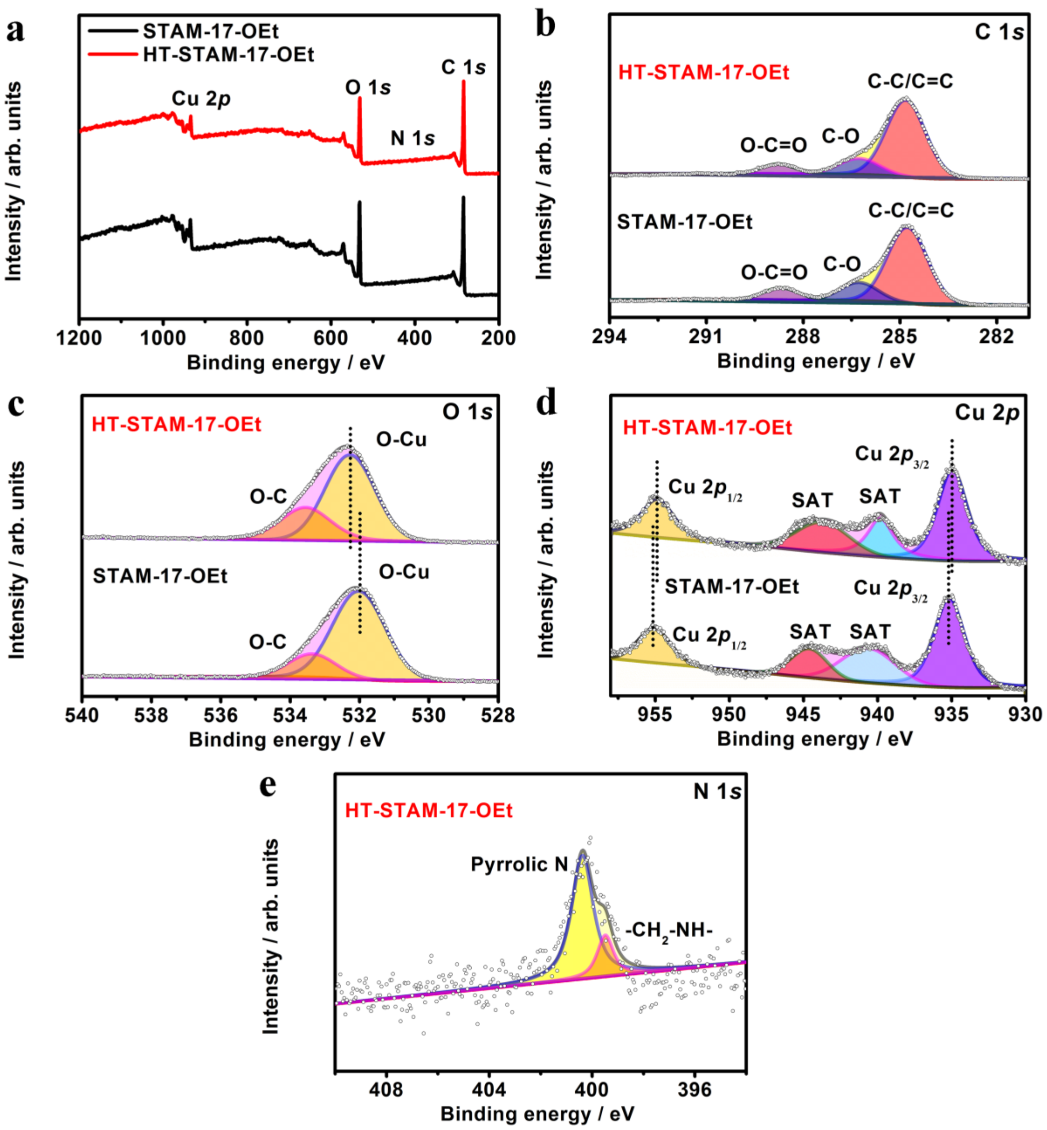

Supplementary Fig 11. (a) XPS results for STAM-17-OEt and HT-STAM-17-OEt. XPS spectra of (b) C 1*s*, (c) O 1*s*, (d) Cu 2*p* for STAM-17-OEt and HT-STAM-17-OEt. (e) XPS spectra of N 1*s* for HT-STAM-17-OEt.

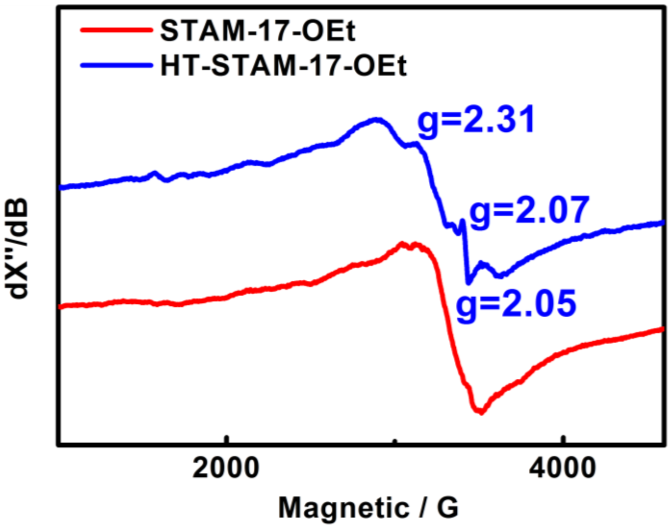

Supplementary Fig 12. Electron paramagnetic resonance (EPR) signals for STAM-17-OEt and HT-STAM-17-OEt.

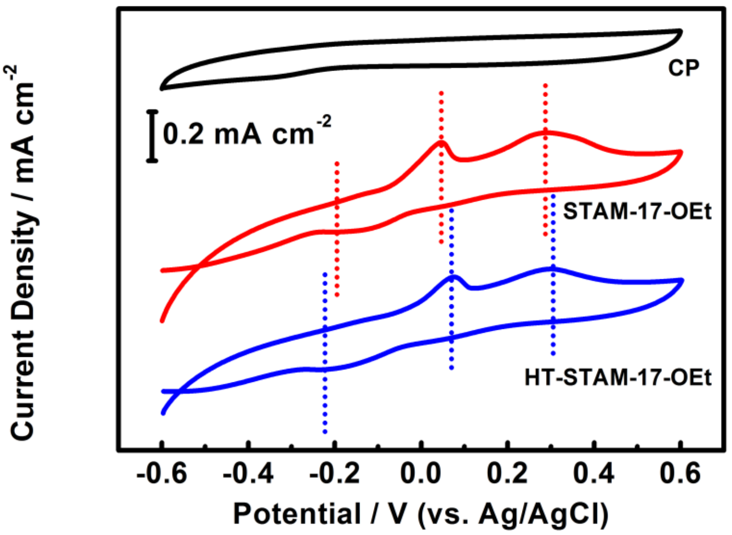

Supplementary Fig 13. CV curves of carbon paper, carbon paper supported STAM-17-OEt, and carbon paper supported HT-STAM-17-OEt in 0.1 M NaCl (pH=7).

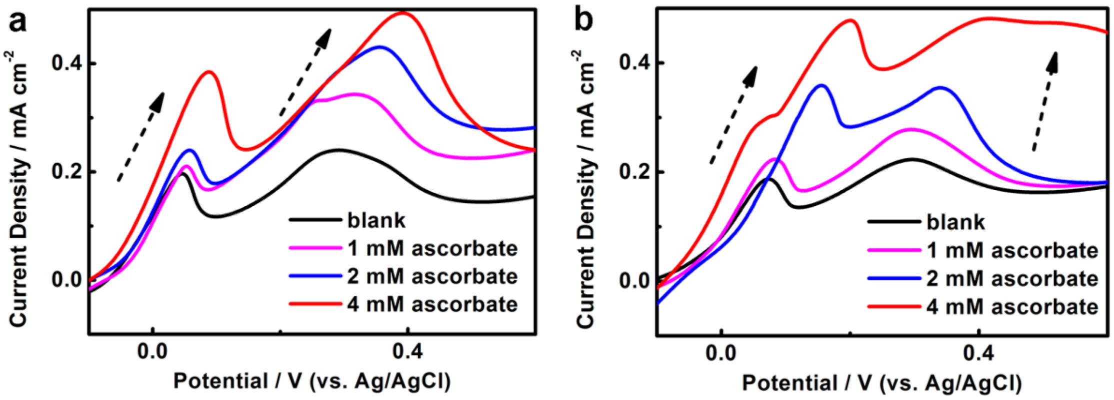

Supplementary Fig 14. CV curves of (a) STAM-17-OEt and (b) HT-STAM-17-OEt in 0.1 M NaCl solution (pH=7) containing 0, 1, 2 and 4 mM ascorbate. Scan rate: 20 mV s^{-1}.

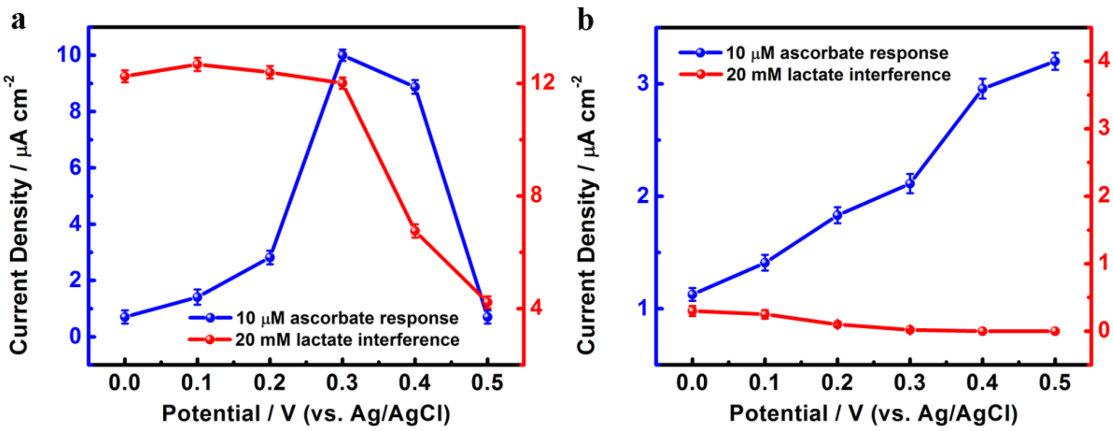

Supplementary Fig 15. The relationship between ascorbate responses, lactate interference and applied potential for (a) STAM-17-OEt and (b) HT-STAM-17-OEt. Data are presented as median values. Relative standard deviation was obtained from three groups of independent samples examined over independent current response test.

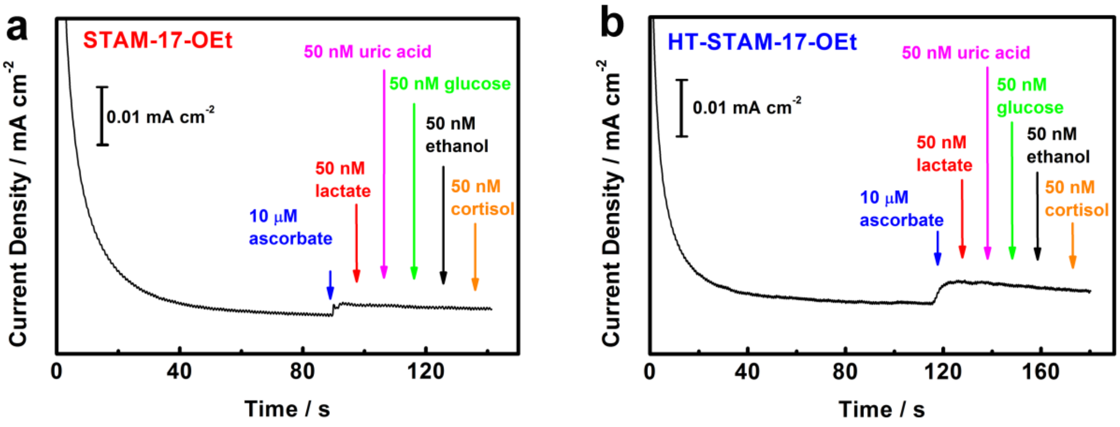

Supplementary Fig 16. (a-b) Amperometry response of STAM-17-OEt and HT-STAM-17-OEt to 10 μM ascorbate with high low interference (50 nM) in 0.1 M NaCl (pH=7) at 0.5 V vs. Ag/AgCl.

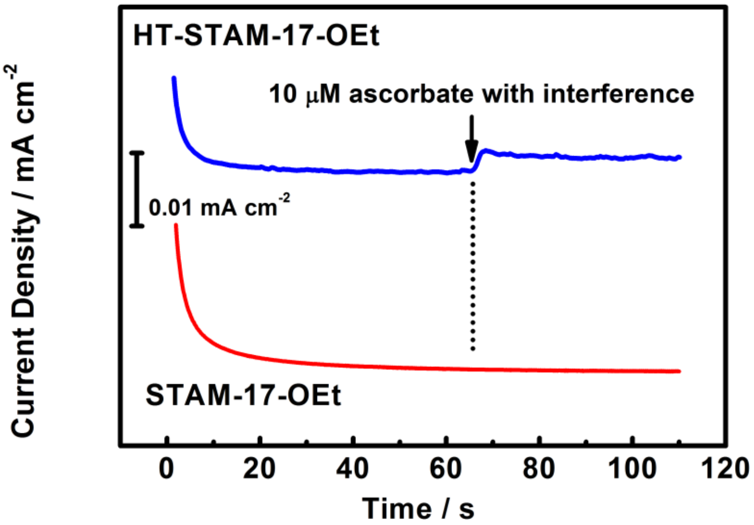

Supplementary Fig 17. Amperometry response of STAM-17-OEt and HT-STAM-17-OEt to 10 μM ascorbate with high concentrated interference in 0.1 M NaCl (pH=7) at 0.5 V. The interference included 20 mM lactate, 10 mM uric acid, 0.2 mM glucose, 22.5 mM ethanol and 0.386 μM cortisol.

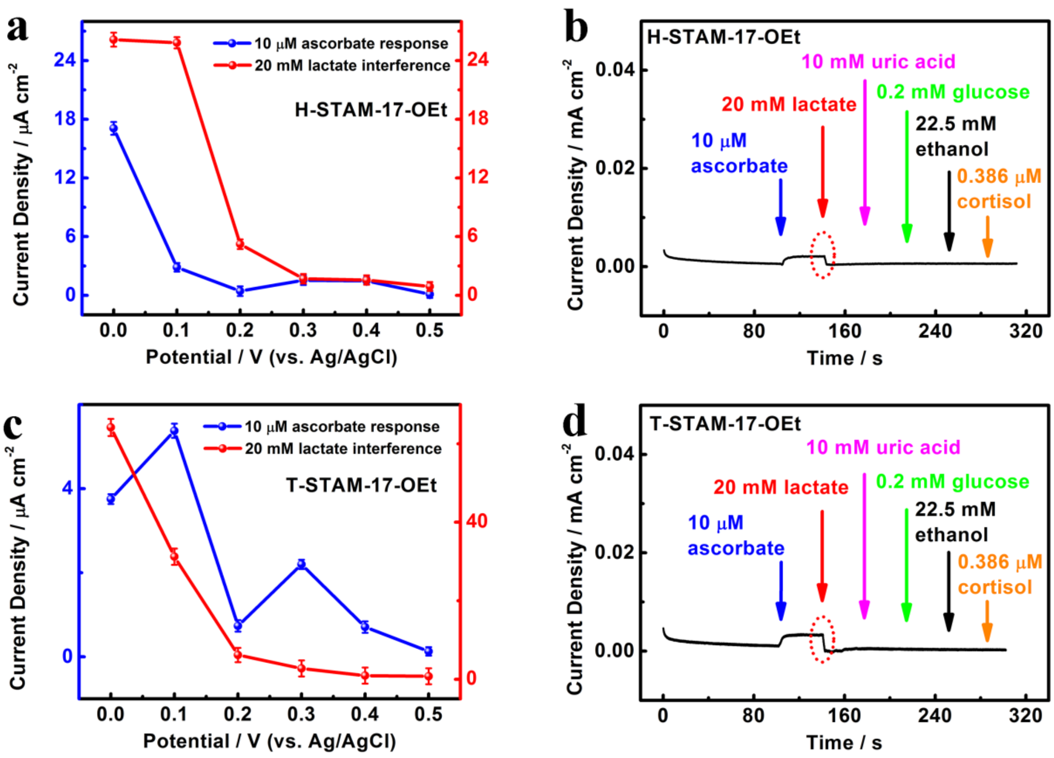

Supplementary Fig 18. (a) The relationship between ascorbate responses, lactate interference and applied potential for H-STAM-17-OEt. (b) Selectivity test of H-STAM-17-OEt. (Applied potential: 0.3 V (vs. Ag/AgCl). (c) The relationship between ascorbate responses, lactate interference and applied potential for T-STAM-17-OEt. (d) Selectivity test of T-STAM-17-OEt. (Applied potential: 0.3 V (vs. Ag/AgCl). Data are presented as median values. Relative standard deviation was obtained from three groups of independent samples examined over independent current response test.

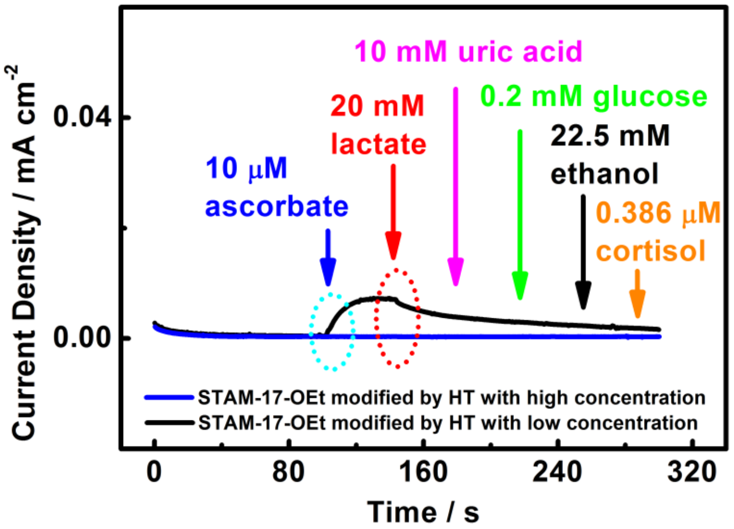

Supplementary Fig 19. Selectivity test of STAM-17-OEt treated with a mixture of tryptophan and histidine (HT) at different concentrations. The high concentrations are 50 mM for tryptophan and 25 mM for histidine. The low concentrations are 0.5 mM for tryptophan and 0.25 mM for histidine. The applied potential is 0.5 V (vs. Ag/AgCl).

Using highly concentrated mixed tryptophan and histidine (50 mM for tryptophan and 25 mM for histidine) to react with STAM-17-OEt would result in great destruction of the structure of STAM-17-OEt. We consider this is due to the strong etching effect of highly concentrated mixed tryptophan and histidine. Such HT-STAM-17-OEt has no electro-oxidation activity to ascorbate or lactate interference because more His and Trp molecules would fully cover the formed structure that is responsible for selectivity. On the other hand, using a low concentration of mixed tryptophan and histidine (0.5 mM for tryptophan and 0.25 mM for histidine) to react with STAM-17-OEt did not destroy STAM-17-OEt, and lactate interference was significant, which was due to almost no amino acids on the surface of STAM-17-OEt. Therefore, the pocket for the specific capture of ascorbate does not form in these two cases. On the other hand, His or Trp individually treated STAM-17-OEt still possess relatively weak lactate interference, indicating that the pocket consisting of solely His or Trp molecules can capture ascorbate, as well as lactate.

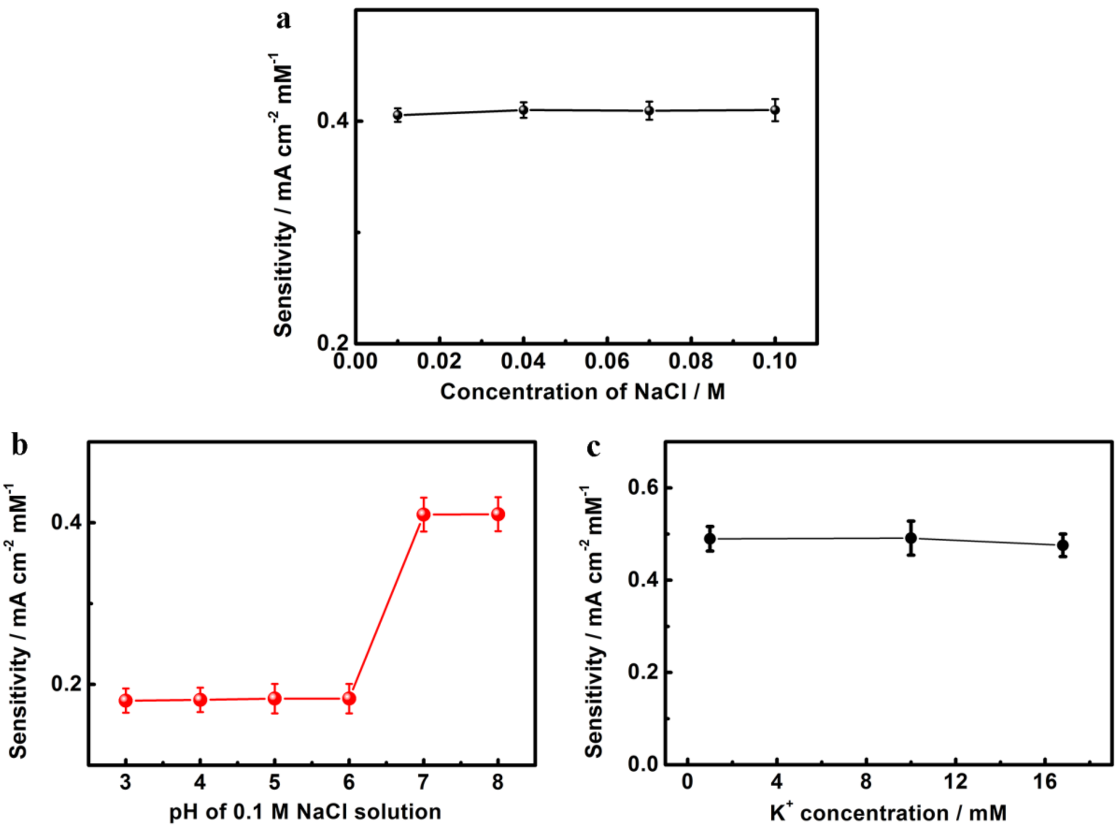

Supplementary Fig 20. Relationship between ascorbate sensitivity of HT-STAM-17-OEt and (a) NaCl concentration (pH=7), (b) pH of NaCl (0.1 M), (C) K^+ concentration in 0.1 M NaCl (pH=7). Data are presented as median values. Relative standard deviation was obtained from three groups of independent samples examined over independent selectivity evaluation.

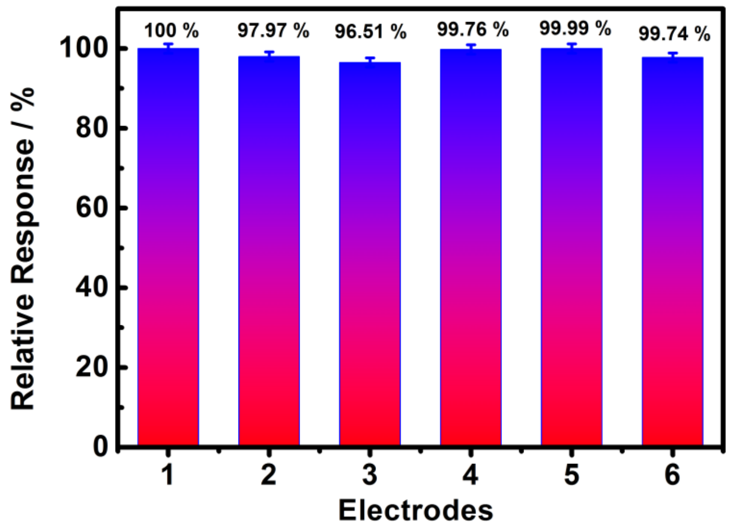

Supplementary Fig 21. Current response of six different HT-STAM-17-OEt-based electrodes to ascorbate in 0.1 M NaCl (pH=7). (Applied potential: 0.5 V (vs. Ag/AgCl). Data are presented as median values Relative standard deviation was obtained from three groups of independent samples examined over independent current response test.

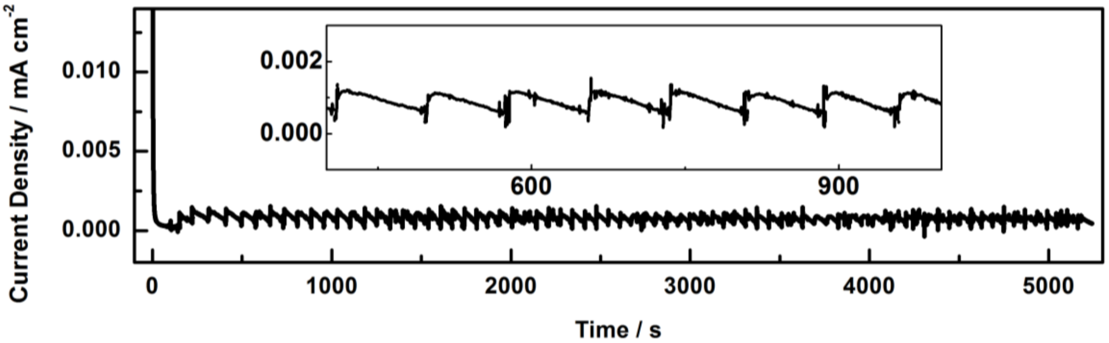

Supplementary Fig 22. Current response of the HT-STAM-17-OEt-based electrode to continuous injection of 5 μM ascorbate in 0.1 M NaCl (pH=7).

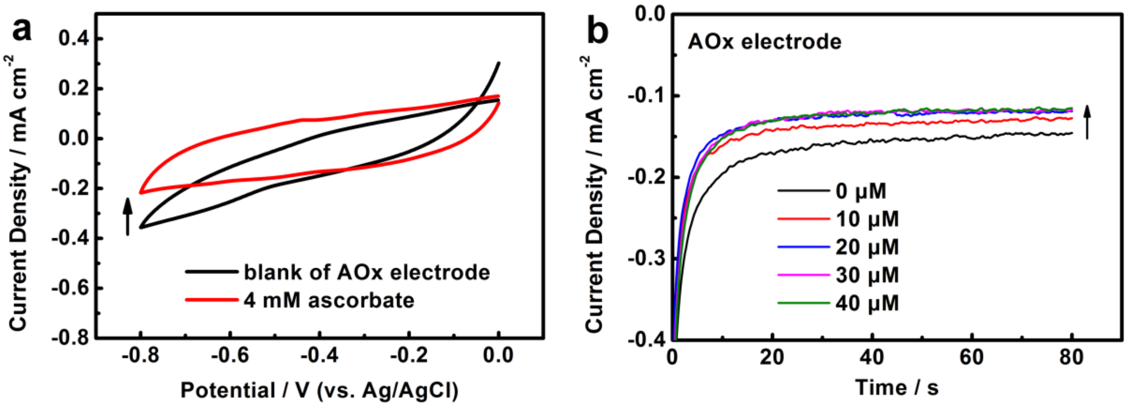

Supplementary Fig 23. (a) CV curves at 20 mV s^{-1} and (b) amperometry response at -0.6 V vs. Ag/AgCl for the AOx-based electrode in 0.1 M NaCl (pH=7).

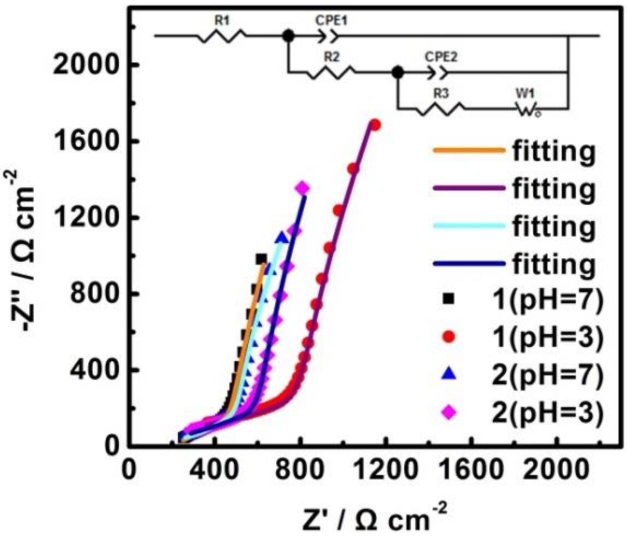

Supplementary Fig 24. EIS results of HT-STAM-17-OEt in different electrolytes with pH values of 3 and 7. 0.1 M NaCl, 0.1 M NaCl with 18.5 mM KCl are denoted as 1 and 2, respectively. The electrode area was 0.23758 cm^{-2}. The inset figure is the equivalent circuit. R1 is the solution resistance, CPE1 is the capacitance of the amino acid layer, R2 is the resistance of the amino acid layer, CPE2 is the capacitance of the STAM-17-OEt surface, and R3 is the charge transfer resistance. w1 (Warburg resistance) is the diffusion resistance.

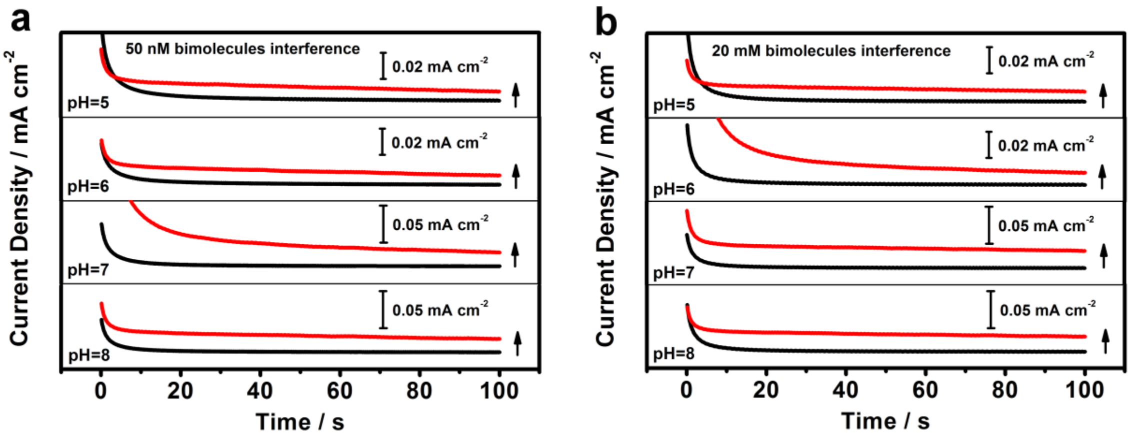

Supplementary Fig 25. Amperometry response of HT-STAM-17-OEt to 50 μM ascorbate at 0.5 V vs. Ag/AgCl in different acid and basic salt solutions (0.1 M NaCl, 20 mM KCl $CaCl_2$ and NH_4Cl) containing (a) 50 nM lactate, uric acid, glucose, ethanol and cortisol and (b) 20 mM lactate, uric acid, glucose, ethanol and cortisol.

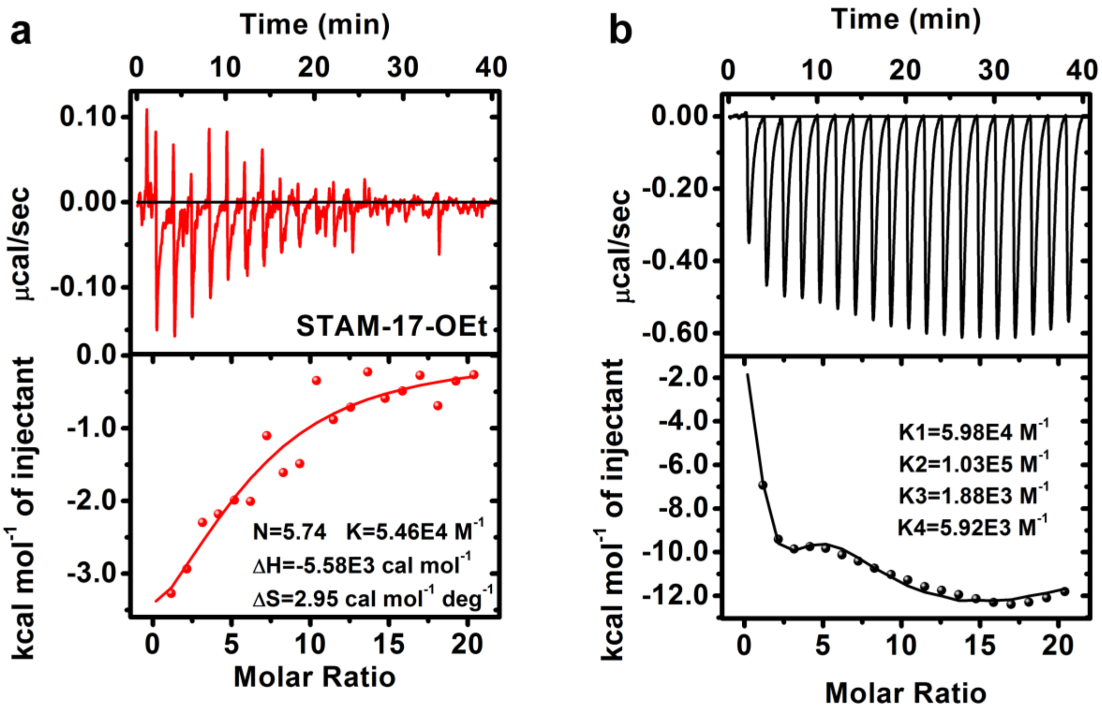

Supplementary Fig 26. ITC curve of ascorbate binding by (a) STAM-17-OEt and (b) ascorbate oxidase.

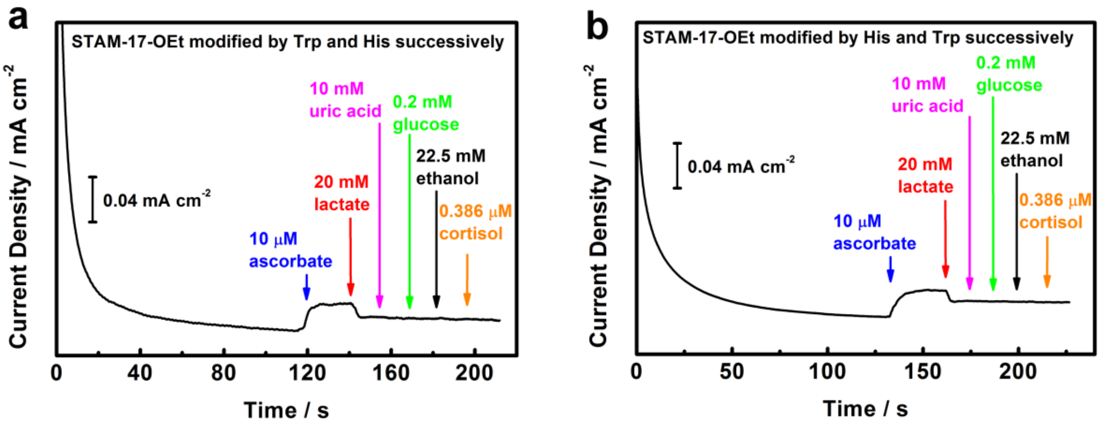

Supplementary Fig 27. Selectivity test of STAM-17-OEt treated with His (2.5 mM) and Trp (5 mM) in different order. (a) Current response of STAM-17-OEt modified by first using 5 mM Trp and then 2.5 mM His. (b) Current response of STAM-17-OEt modified by first using 2.5 mM His and then 5 mM Trp. The applied potential is 0.5 V (vs. Ag/AgCl).

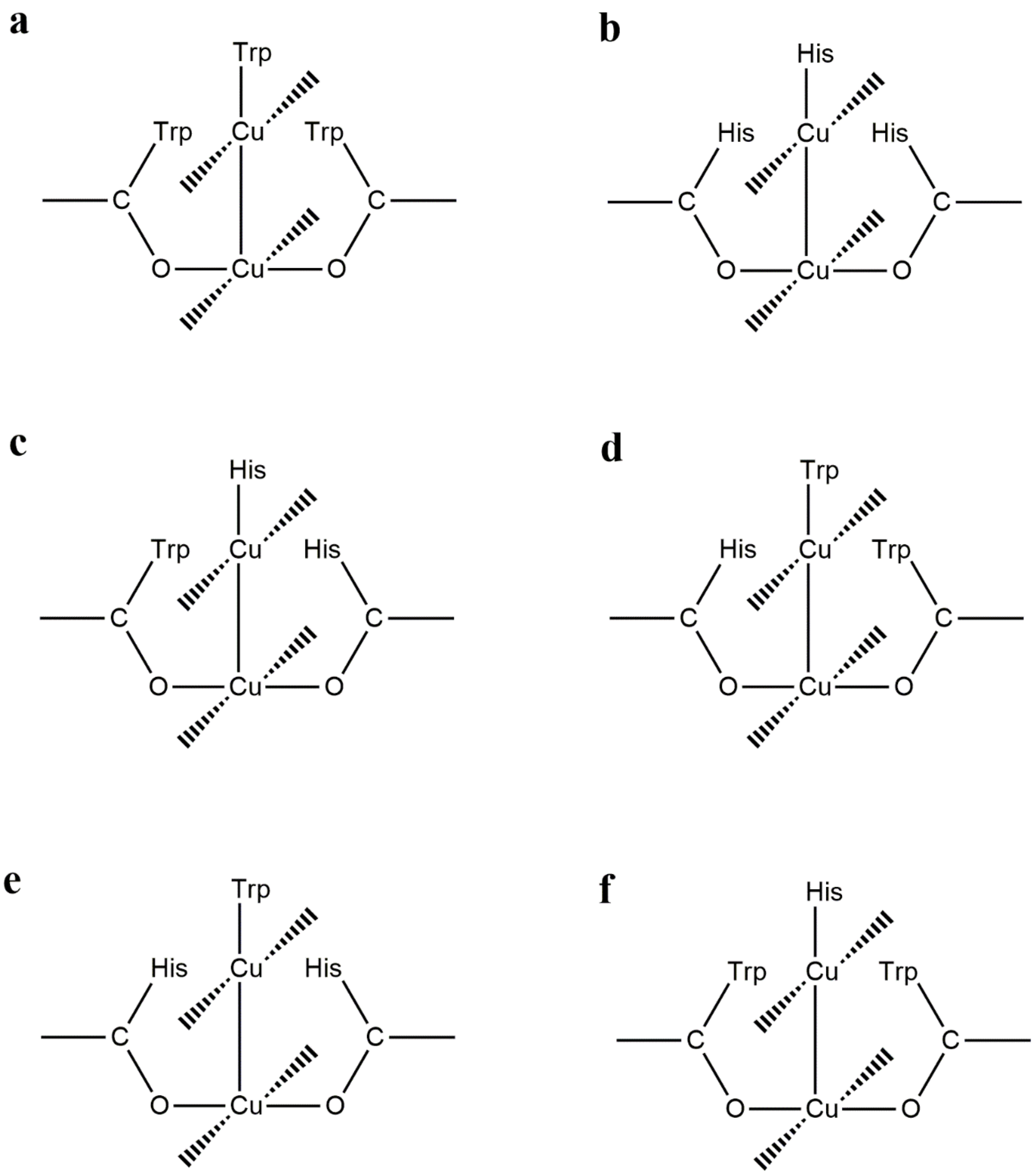

Supplementary Fig 28. Six possible structures formed by Trp and His on the surface Cu paddle wheel. (a) Three Trp are connected to Cu along the axial direction and both sides. (b) Three His are connected to Cu along the axial direction and both sides. (c) One His is connected to Cu along the axial direction and one His and Trp are connected to Cu on both sides. (d) One Trp is connected to Cu along the axial direction and one His and Trp are connected to Cu on both sides. (e) One Trp is connected to Cu along the axial direction and two His are connected to Cu on both sides. (f) One His is connected to Cu along the axial direction and two Trp are connected to Cu on both sides.

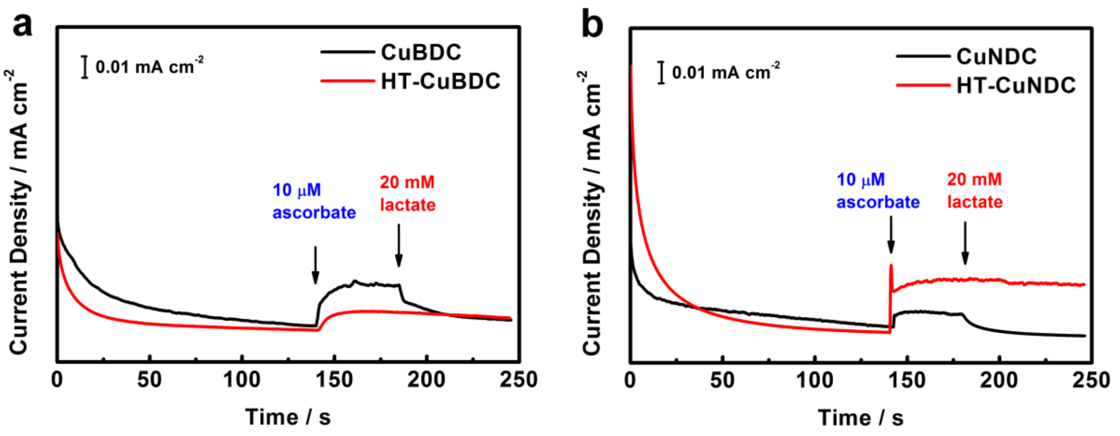

Supplementary Fig 29. Selectivity test of (a) CuBDC and (b) CuNDC treated with 5 mM Trp/2.5 mM His. The applied potential is 0.5 V (vs. Ag/AgCl). CuBDC is composed of benzene-1,4-dicarboxylic acid (BDC) and Cu ions. CuNDC is composed of 1,4-naphthalenedicarboxylic acid (NDC) and Cu ions.

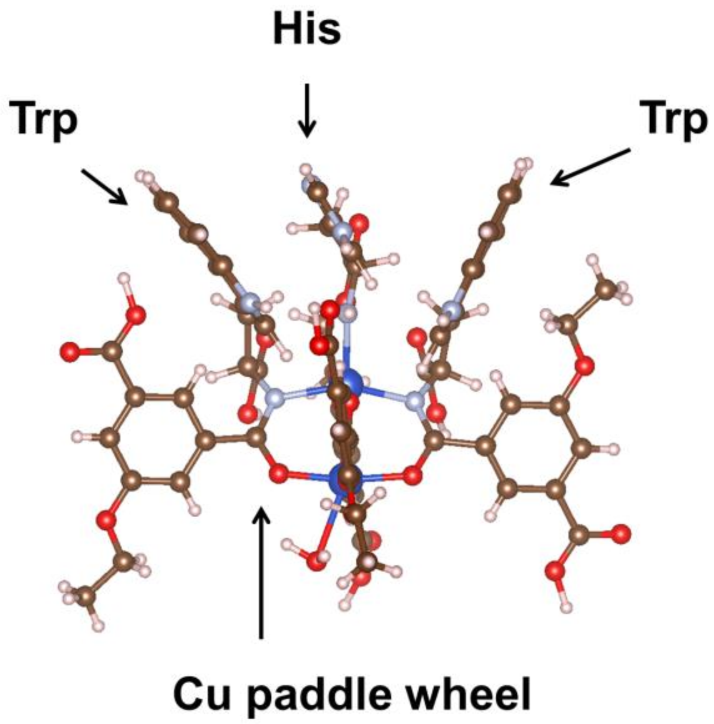

Supplementary Fig 30. Geometric optimized structure f.

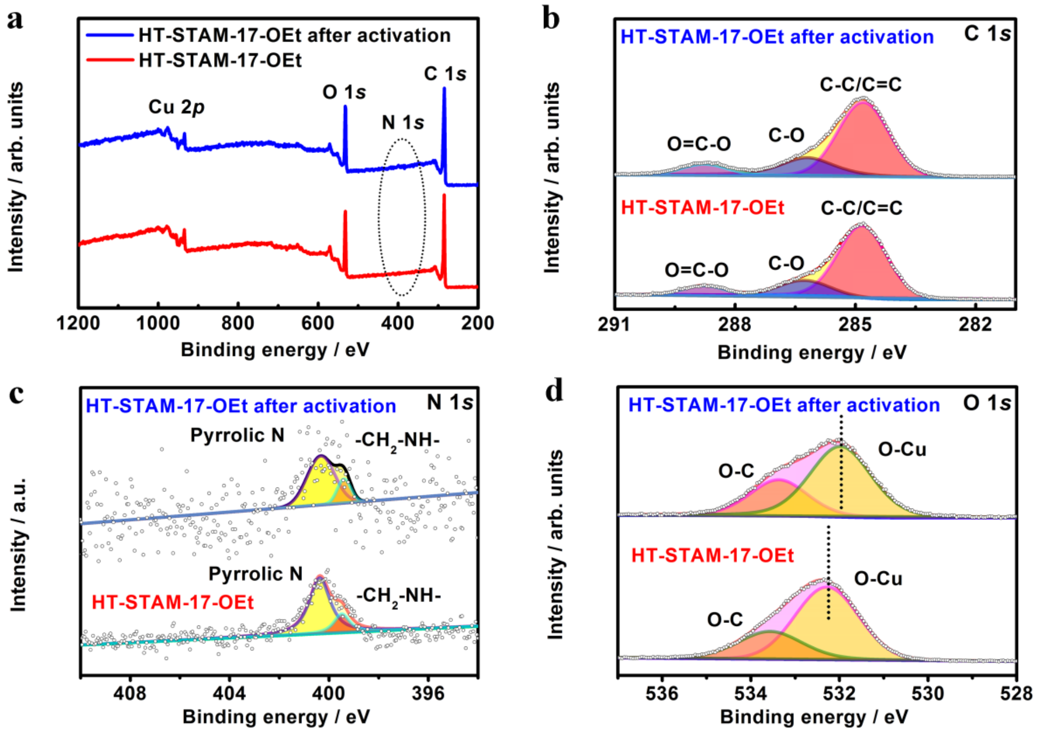

Supplementary Fig 31. (a) XPS results for HT-STAM-17-OEt and HT-STAM-17-OEt after activation. XPS spectra of (b) C 1*s*, (c) N 1*s*, (d) O 1*s* for HT-STAM-17-OEt and HT-STAM-17-OEt after activation.

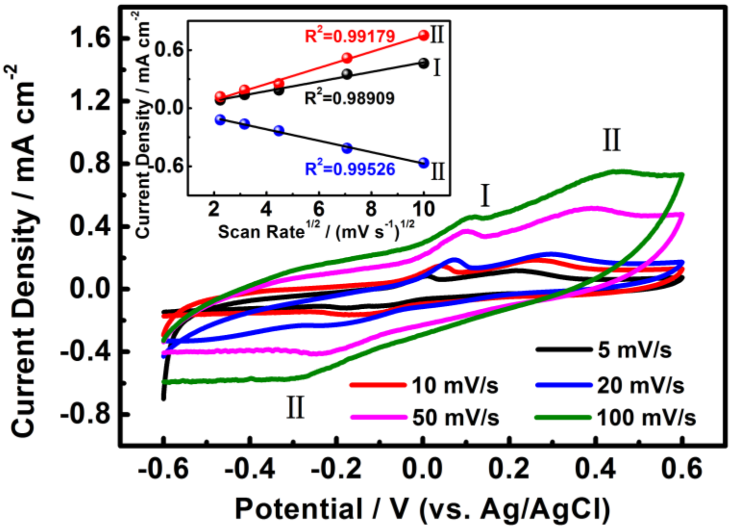

Supplementary Fig 32. CV curves of carbon-supported HT-STAM-17-OEt in 0.1 M NaCl (pH=7) with different scan rates. The inset is the relationship between the peak currents and the square root of the scan rates.

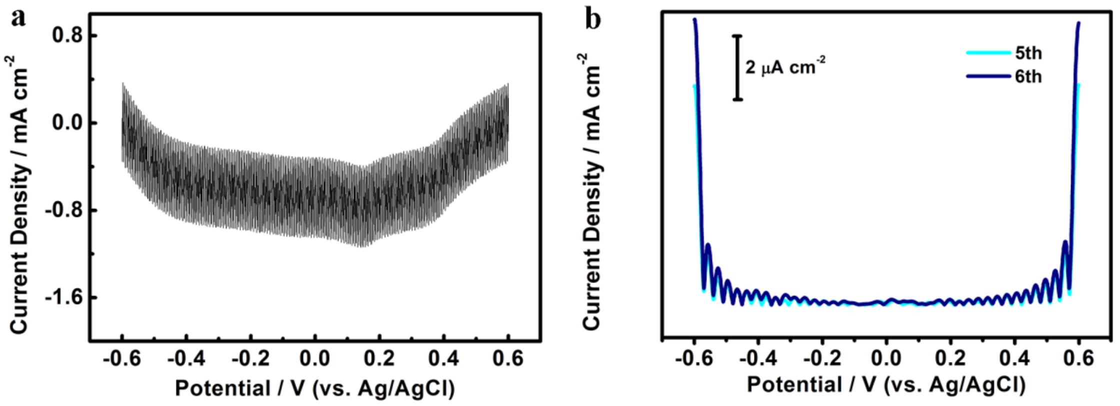

Supplementary Fig 33. (a) FT ac voltammogram result of HT-STAM-17-OEt in 0.1 M NaCl (pH=7) and (b) corresponding fifth and sixth harmonic components of FTac voltammograms. The frequency and amplitude are 9 Hz and 80 mV, respectively.

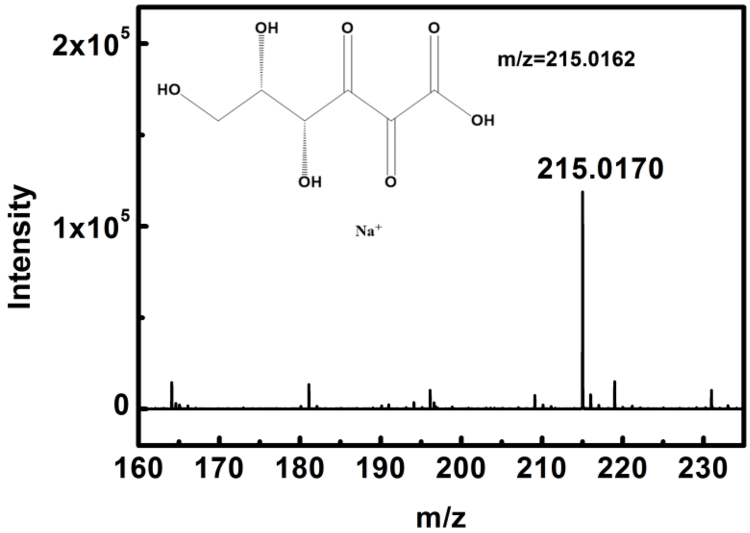

Supplementary Fig 34. High resolution mass spectrometry (HRMS) of ascorbate oxidation product by HT-STAM-17-OEt.

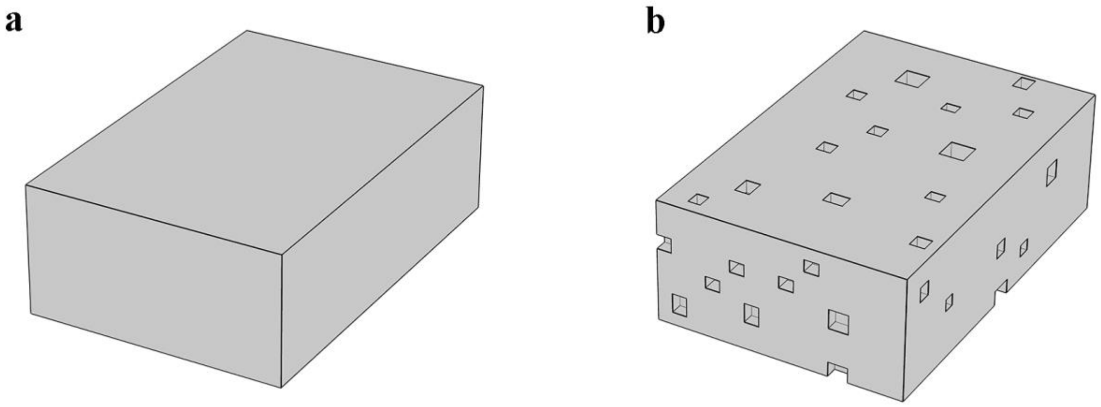

Supplementary Fig 35. 3D geometry model of (a) STAM-17-OEt and (b) HT-STAM-17-OEt crystal.

Supplementary Tab 1. Elements mass percentage for HT-STAM-17-OEt by XPS characterization.

O	C	Cu	N
22.7%	61.7%	14.6%	0.9%

Supplementary Tab 2. EIS fitting result for HT-STAM-17-OEt in different electrolytes.

	0.1 M NaCl (pH=7)	0.1 M NaCl (pH=3)	0.1 M NaCl with 18.5 mM KCl (pH=7)	0.1 M NaCl with 18.5 mM KCl (pH=3)
R1 (Ω cm^{-2})	203.7629	99.2087	149.8863	156.9997
CPE1-T	8.3277E-5	1.4166E-4	2.3274E-4	1.0594E-4
CPE1-P	0.60034	0.42802	0.45376	0.50479
R2 (Ω cm^{-2})	69.6187	0.5421	164.8708	0.7419
CPE2-T (F cm^{-2})	3.7099E-13	2.7443E-17	8.5192E-10	3.5575E-13
CPE2-P	0.74183	0.87276	0.031311	0.5195
R3 (Ω cm^{-2})	143.9936	143.9936	105.2277	377.0940
W1-R (Ω cm^{-2})	467.2110	467.2110	657.4627	607.7953
W1-T (F cm^{-2})	0.001818	0.001818	0.002210	0.001778
W1-P	0.47629	0.47629	0.49485	0.48174

Supplementary Tab 3. Total energies for the calculated structures.

Structure	Total energy (eV)
Trp/His functionalized Cu paddle wheel	-222481.25557
ascorbate	-18635.98838
lactate	-9334.23630
Trp/His functionalized Cu paddle wheel-ascorbate	-241118.02299
Trp/His functionalized Cu paddle wheel-lactate	-231813.74826

Supplementary Tab 4. Boundary condition parameters for simulating the current density on micro/nanostructures of STAM-17-OEt and HT-STAM-17-OEt.

i_o (A m^{-2})	α_a	α_c	C_R	C_O	P (V)
1	0.5	0.5	1	1000	0.5

In Table S4, i_o is the exchange current density, and α_a and α_c are the anode and cathode transfer coefficients, respectively. C_R and C_O are reduction and oxidation species constants, respectively. P is the external potential. By adjusting the values of C_O and P, the dimensionless current densities at different overpotentials can be displayed.

Supplementary References

1 T. Guinovart, G. A. Crespo, F. X. Rius, F. J. Andrade, *Anal. Chim. Acta*. ***821***, 72-80 (2014).
