# Supplementary material for: Natural oxidase-mimicking copper-organic frameworks for targeted identification of ascorbate in sensitive sweat sensing
Source: Nat Commun. 2023 Jan 5;14:69. doi: 10.1038/s41467-022-35721-4 (PMC9814535; doi:10.1038/s41467-022-35721-4)
Supplement: Supplementary file 3 — Reporting Summary [file 41467_2022_35721_MOESM3_ESM.pdf]

nature portfolio

Corresponding author(s): DBPR NCOMMS-22-28757B

Last updated by author(s): 2022.12.12

Reporting Summary

Nature Portfolio wishes to improve the reproducibility of the work that we publish. This form provides structure for consistency and transparency in reporting. For further information on Nature Portfolio policies, see our Editorial Policies and the Editorial Policy Checklist.

Statistics

For all statistical analyses, confirm that the following items are present in the figure legend, table legend, main text, or Methods section.

n/a	Confirmed	
☐	☒	The exact sample size (*n*) for each experimental group/condition, given as a discrete number and unit of measurement
☐	☒	A statement on whether measurements were taken from distinct samples or whether the same sample was measured repeatedly
☒	☐	The statistical test(s) used AND whether they are one- or two-sided<br>*Only common tests should be described solely by name; describe more complex techniques in the Methods section.*
☒	☐	A description of all covariates tested
☒	☐	A description of any assumptions or corrections, such as tests of normality and adjustment for multiple comparisons
☐	☒	A full description of the statistical parameters including central tendency (e.g. means) or other basic estimates (e.g. regression coefficient) AND variation (e.g. standard deviation) or associated estimates of uncertainty (e.g. confidence intervals)
☒	☐	For null hypothesis testing, the test statistic (e.g. *F*, *t*, *r*) with confidence intervals, effect sizes, degrees of freedom and *P* value noted<br>*Give P values as exact values whenever suitable.*
☒	☐	For Bayesian analysis, information on the choice of priors and Markov chain Monte Carlo settings
☒	☐	For hierarchical and complex designs, identification of the appropriate level for tests and full reporting of outcomes
☒	☐	Estimates of effect sizes (e.g. Cohen's *d*, Pearson's *r*), indicating how they were calculated

Our web collection on statistics for biologists contains articles on many of the points above.

Software and code

Policy information about availability of computer code

Data collection	Materials Studio (8.0) was used for density functional theory calculation. Comsol Multiphysics (5.3a) was used for finite element analysis. And they are mentioned in the manuscript.
Data analysis	We have not used code in this study.

For manuscripts utilizing custom algorithms or software that are central to the research but not yet described in published literature, software must be made available to editors and reviewers. We strongly encourage code deposition in a community repository (e.g. GitHub). See the Nature Portfolio guidelines for submitting code & software for further information.

Data

Policy information about availability of data

All manuscripts must include a data availability statement. This statement should provide the following information, where applicable:

- Accession codes, unique identifiers, or web links for publicly available datasets
- A description of any restrictions on data availability
- For clinical datasets or third party data, please ensure that the statement adheres to our policy

The data that support the findings of this study are available from the corresponding author upon request

Human research participants

Policy information about studies involving human research participants and Sex and Gender in Research.

Reporting on sex and gender	Our study does not include sex/gender related research finding. In our work, the electrochemical test of sweat is only to verify the sensing functionality of the materials rather than to evaluate the healthy status of individual according to the sensing result, thus sex and/or gender was not included on the title and abstract.
Population characteristics	The volunteer is 29 years old and healthy. In this work, individual health status related study are not included.
Recruitment	Sweat samples were obtained from one healthy volunteer (male) who provided informed consent, and confirm that they complied with all ethical regulations. Self-selection bias or other bias are not present In this work because sweat testing is only to verify the sensing function of materials, rather than including study content about evaluating the healthy status.
Ethics oversight	Ethics Committee of Clinical Drug Trials of Huazhong University of Science and Technology confirmed that protocol approval is not required for only using sweat to evaluate the materials function.

Note that full information on the approval of the study protocol must also be provided in the manuscript.

Field-specific reporting

Please select the one below that is the best fit for your research. If you are not sure, read the appropriate sections before making your selection.

- [x] Life sciences
- [ ] Behavioural & social sciences
- [ ] Ecological, evolutionary & environmental sciences

For a reference copy of the document with all sections, see nature.com/documents/nr-reporting-summary-flat.pdf

Life sciences study design

All studies must disclose on these points even when the disclosure is negative.

Sample size	In this work, aiming to verify the materials sensing function, collecting some sweat from one volunteer is sufficient.
Data exclusions	No data exclusions in this study.
Replication	The ascorbate sensing function of materials has been successfully evaluated by repeated sweat test with three replications.
Randomization	Sweat is randomly collected from the body part of volunteer.
Blinding	As it does not involve health assessment, blinding study is not related to materials sensing function evaluation.

Reporting for specific materials, systems and methods

We require information from authors about some types of materials, experimental systems and methods used in many studies. Here, indicate whether each material, system or method listed is relevant to your study. If you are not sure if a list item applies to your research, read the appropriate section before selecting a response.

Materials & experimental systems

n/a	Involved in the study	
x		Antibodies
x		Eukaryotic cell lines
x		Palaeontology and archaeology
x		Animals and other organisms
x		Clinical data
x		Dual use research of concern

Methods

n/a	Involved in the study	
x		ChIP-seq
x		Flow cytometry
x		MRI-based neuroimaging
